# Metabolic Signatures of *Cryptosporidium* *parvum*-Infected HCT-8 Cells and Impact of Selected Metabolic Inhibitors on *C. parvum* Infection under Physioxia and Hyperoxia

**DOI:** 10.3390/biology10010060

**Published:** 2021-01-15

**Authors:** Juan Vélez, Zahady Velasquez, Liliana M. R. Silva, Ulrich Gärtner, Klaus Failing, Arwid Daugschies, Sybille Mazurek, Carlos Hermosilla, Anja Taubert

**Affiliations:** 1 Biomedical Research Center Seltersberg, Institute of Parasitology, Justus Liebig University-Giessen, Schubert Str. 81, 35392 Giessen, Germany; zahady.velasquez@vetmed.uni-giessen.de (Z.V.); liliana.silva@vetmed.uni-giessen.de (L.M.R.S.); carlos.r.hermosilla@vetmed.uni-giessen.de (C.H.); anja.taubert@vetmed.uni-giessen.de (A.T.); 2 Institute of Veterinary Physiology and Biochemistry, Justus Liebig University-Giessen, Frankfurter Str. 100, 35392 Giessen, Germany; sybille.mazurek@vetmed.uni-giessen.de; 3 Institute of Anatomy and Cell Biology, Justus Liebig University-Giessen, Aulweg 123, 35392 Giessen, Germany; ulrich.gaertner@anatomie.med.uni-giessen.de; 4 Unit for Biomathematics and Data Processing, Justus Liebig University-Giessen, Frankfurter Str. 95, 35392 Giessen, Germany; klaus.failing@vetmed.uni-giessen.de; 5 Institute of Parasitology, University of Leipzig, An den Tierkliniken 35, 04103 Leipzig, Germany; daugschies@vetmed.uni-leipzig.de

**Keywords:** *Cryptosporidium parvum*, cryptosporidiosis, hyperoxia, physioxia, glycolysis, glutaminolysis

## Abstract

**Simple Summary:**

*Cryptosporidium parvum* is one of the causal agents of cryptosporidiosis. This water-borne disease is responsible for around one million human deaths every year and the only approved anti-cryptosporidial drug for humans, i.e., nitazoxanide, lacks efficacy in immunocompromised patients. We here present, to the best of our knowledge, the first analyses of *C. parvum* impact on the metabolism of its host-cells under physiological oxygen conditions. Moreover, based on the here presented metabolic evidence, we blocked specific metabolic pathways in order to find new anti-cryptosporidial targets. Our findings besides confirming glycolysis as anti-cryptosporidial target prove glutaminolysis and lactate release as necessary for parasite replication.

**Abstract:**

*Cryptosporidium parvum* is an apicomplexan zoonotic parasite recognized as the second leading-cause of diarrhoea-induced mortality in children. In contrast to other apicomplexans, *C.* *parvum* has minimalistic metabolic capacities which are almost exclusively based on glycolysis. Consequently, *C. parvum* is highly dependent on its host cell metabolism. In vivo (within the intestine) infected epithelial host cells are typically exposed to low oxygen pressure (1–11% O_2_, termed physioxia). Here, we comparatively analyzed the metabolic signatures of *C. parvum*-infected HCT-8 cells cultured under both, hyperoxia (21% O_2_), representing the standard oxygen condition used in most experimental settings, and physioxia (5% O_2_), to be closer to the in vivo situation. The most pronounced effect of *C. parvum* infection on host cell metabolism was, on one side, an increase in glucose and glutamine uptake, and on the other side, an increase in lactate release. When cultured in a glutamine-deficient medium, *C. parvum* infection led to a massive increase in glucose consumption and lactate production. Together, these results point to the important role of both glycolysis and glutaminolysis during *C. parvum* intracellular replication. Referring to obtained metabolic signatures, we targeted glycolysis as well as glutaminolysis in *C. parvum*-infected host cells by using the inhibitors lonidamine [inhibitor of hexokinase, mitochondrial carrier protein (MCP) and monocarboxylate transporters (MCT) 1, 2, 4], galloflavin (lactate dehydrogenase inhibitor), syrosingopine (MCT1- and MCT4 inhibitor) and compound 968 (glutaminase inhibitor) under hyperoxic and physioxic conditions. In line with metabolic signatures, all inhibitors significantly reduced parasite replication under both oxygen conditions, thereby proving both energy-related metabolic pathways, glycolysis and glutaminolysis, but also lactate export mechanisms via MCTs as pivotal for *C. parvum* under in vivo physioxic conditions of mammals.

## 1. Introduction

*Cryptosporidium parvum* is one of the most important causal agents of human and animal cryptosporidiosis [1,2,3,4,5]. This zoonotic parasite (subphylum Apicomplexa) is considered the second-leading aetiology of diarrhoea-related mortality in children [4,6,7], and responsible for almost a million human deaths each year [8,9]. Moreover, the only approved drug in humans against cryptosporidiosis, i.e., nitazoxanide, is neither effective in affected children nor in patients with impaired immune systems [3,10,11,12]. Therefore, novel effective anti-cryptosporidial therapies are urgently needed, and cryptosporidiosis still represents a challenge for public health worldwide.

*C. parvum* is a fast-replicating obligate intracellular protozoan with minimalistic metabolic capabilities as a result of a reductive evolution process. This is clearly reflected in its minute genome, merely comprising 9.1 Mb in eight chromosomes compared to 23 Mb in 14-chromosomes of *Plasmodium falciparum* [13] or other closely related apicomplexans (e.g., *Eimeria*, *Toxoplasma*, *Neospora*, *Sarcocystis*). Consequently, *C. parvum* streamlined metabolic repertoire lacks tricarboxylic acid cycle, oxidative phosphorylation, de novo pyrimidine, amino acid and cholesterol biosynthesis thereby mainly relying on glycolysis for energy production [13,14,15,16,17,18]. Likewise, *C. parvum* possesses an extensive pool of genes encoding for families of putative sugar and amino acid transporters [13,14].

Interestingly, *Cryptosporidium* spp. have emerged from a gregarines’ common ancestor which shows a distinct primitive nutritional behaviour known as myzocytosis or “cellular vampirism” by using a feeder organelle for piercing prey cell membranes and sucking out captured nutrients [15,19,20,21,22]. In fact, such a predatory strategy could represent the starting point in the transition to a parasitic lifestyle in the earliest apicomplexan ancestors as previously postulated [15,21,22]. Reminiscences of such predatory past are the epicellular, i.e., intracellular but extracytoplasmatic parasite localization, and the unique feeder organelle of *Cryptosporidium*. Successful myzocytotic feeding behaviour is also reported for closely related dinoflagellates and archigregarines [21,22]. Thus, *Cryptosporidium* spp. attachment and feeding behaviour furnish these protozoans to scavenge host cell nutrients to support their fast and energy-demanding intracellular replication.

Referring to metabolic *C. parvum*-host cell-interactions, gene expression-based evidences showed a down-regulation of host-parasite homologous genes in host glycolysis/gluconeogenesis pathways (e.g., phosphofructokinase, aldolase, GAPDH, among others) while host-exclusive genes were upregulated, thereby suggesting a parasite-derived direct competition for metabolic substrates [23]. Gas-chromatography-mass spectrometry (GC-MS)-based studies revealed qualitative and quantitative differences in the faecal metabolite profile of infected and non-infected humans [24] as well as infected and control mice [25]. Interestingly, such faecal metabolite profiles revealed significant higher metabolite concentrations in human infected samples while murine infected samples showed significant lower metabolite concentrations, pointing out that differences in the metabolic impact depend on the host. More recently, a ^1^H-nuclear magnetic resonance (NMR)-based study confirmed differences in host cell metabolism of *Cryptosporidium*-infected cells both in vitro (COLO-680N cell line) as well as in vivo (murine ileum) [26]. However, increased lactate levels were detected in both COLO-680N and murine ileum [26]. The lactate may derive from increased glucose or amino acid degradation, i.e., glutamine, in *C. parvum*-infected host cells. Several genomic and biochemical evidences confirmed the dependence of *Cryptosporidium* on glycolysis as main energy source [8,13,14,17,27]. Likewise, different studies demonstrated that pharmacological blockage of glycolysis inhibits parasite replication [27,28,29,30], thereby suggesting this metabolic pathway as a promising anti-cryptosporidial target.

Like *Cryptosporidium*, several other apicomplexan parasites, such as *Toxoplasma gondii*, showed glucose-related growth dependence [31,32,33]. Of note, some of these parasites also revealed the capability to compensate low glucose availability by increased glutamine uptake [31,32]. So far, a similar metabolic plasticity has not been described for *C. parvum* and—to the best of our knowledge—there are currently no studies in literature reporting glutaminolysis as a potential anti-cryptosporidial target.

Overall, zoonotic-relevant *C. parvum* is known as a microaerophilic/anaerobic parasite [22,34,35] which naturally resides in the small intestinal epithelium of various mammal species, including humans [36,37]. Of note, the human intestinal epithelium represents one of the largest body organ surfaces and constitutes 40 m^2^ of interface/interaction area with the external environment and commensal and pathogenic organisms [38], rendering this unique organ into a very particular environment with its own special physiological conditions. Thus, oxygen delivery and consumption in the intestine generates a unique sub-micromolar oxygen partial pressure (pO_2_), known as “tissue normoxia” or “physioxia” [39,40]. The consideration of inherent physioxic conditions has gained great importance, especially since several studies proved the pivotal role of oxygen conditions on metabolism at the cellular level [41,42]. Obviously, metabolic reactions strongly depend on current O_2_ conditions. Thus, oxidative phosphorylation is the main energy-regenerating process at high oxygen supply while glycolysis is the main cellular energy source under hypoxic conditions. Besides glycolysis, glutaminolysis represents another important energy source, especially in fast proliferating cells, such as intestinal epithelial cells and tumor cells [43,44,45].

Considering the necessity to evaluate the metabolic impact of *C. parvum* infections at more realistic micromolar oxygen conditions to better simulate the intestinal in vivo situation, we here analyzed the influence of *C. parvum* on host cellular glycolytic and glutaminolytic functions under both physioxic (5%) and hyperoxic (21%) O_2_ concentrations. Based on these results we studied efficacies of selected metabolic inhibitors on parasite infection rates under physioxia- and hyperoxia conditions in vitro.

## 2. Materials and Methods

### 2.1. Host Cell Culture

Permanent HCT-8 cells (ATCC-CCL-244, LGC Standars) were maintained at 37 °C and 5% CO_2_ using sterile RPMI 1640 cell culture medium (R0883, Sigma-Aldrich, Darmstadt, Germany) supplemented with 0.3 g/L L-glutamine (Sigma-Aldrich, Darmstadt, Germany), 10% fetal bovine serum (FBS; S0115, Biochrom AG, Berlin, Germany), 100 UI penicillin and 0.1 mg streptomycin/mL (both Sigma-Aldrich).

HCT-8 cells were seeded at a density of 1 × 10^5^ cells/well into 24-well plastic tissue plates (Eppendorf, Hamburg, Germany) previously treated with 40 µg/mL fibronectin (F1141, Sigma-Aldrich, Darmstadt, Germany). Cells were grown under two different oxygen concentrations, 5% and 21% O_2_, thereby simulating both physioxic conditions of intestine in vivo [17,40] and hyperoxic ones, applied in most *C. parvum*-related studies. Physioxic cultures were performed at 5% O_2_ in a physiological cell culture workstation (InvivO_2_^®^ 400, Ruskinn, Vienna, Austria), while hyperoxic cultures (21% O_2_) used a standardized incubator (Heracell 240i, Thermo Scientific, Langenselbold, Germany). Cell culture medium for physioxic conditions was placed into the workstation for at least 12 h prior to experimentation and shaken manually on a regular basis to allow oxygen equilibration as reported elsewhere [46]. Cell medium in both experimental settings was changed every two days.

### 2.2. Parasites

*Cryptosporidium parvum* oocysts were obtained from experimentally infected calves kept at the large animal stable facility of the Institute of Parasitology, Faculty of Veterinary Medicine, University of Leipzig, Germany, as reported elsewhere [47]. The *C. parvum* strain used here belonged to the subtype 60-kDa glycoprotein (gp60) IIaA15G2RI, which is the most commonly zoonotic subtype to be found in Germany as well as in most other industrialized countries [48,49,50,51]. Oocyst stocks were stored in sterile phosphate buffered saline (PBS, pH 7.4) supplemented with 100 UI penicillin and 0.1 mg streptomycin/mL (P4333, Sigma-Aldrich, Darmstadt, Germany) at 4 °C for a maximum of three months to guarantee infectivity of sporozoites. The above-mentioned oocyst conservation medium was replaced every month as previously described (15).

### 2.3. Host Cell Infection

Sporulated oocysts of *C. parvum* (7 × 10^6^) were pelleted at 5000× *g* for 5 min at 4 °C. Thereafter, sporozoite excystation was induced by supplementation of acidified (pH 2.0) and sterile pre-warmed (37 °C) 1× Hank’s Balanced Salt Solution (HBSS, Sigma-Aldrich, Darmstadt, Germany) for 10 min at 37 °C. Thereafter, free-released *C. parvum*-sporozoites were pelleted (5000× *g* for 5 min) and incubated in non-acidified 1× HBSS for 10 min at 37 °C. Following a final centrifugation step (5000× *g*, 5 min), cells were re-suspended in sterile RPMI 1640 cell culture medium supplement with 0.3 g/L L-glutamine, 10% FBS, 100 UI penicillin and 0.1 mg streptomycin/mL (all Sigma-Aldrich, Darmstadt, Germany). Freshly released and motile sporozoites were added to HCT-8 cell layers for 3 h, thereafter cell layers were washed thrice to remove free sporozoites and oocysts remnants [52], and fresh cell culture medium was added.

### 2.4. Live Cell 3D-Holotomography and Scanning Electron Microscopy (SEM) Analyses of C. parvum-Infected Host Cells

Intracellular development of *C. parvum*-infected HCT-8 cells was illustrated by live cell 3D-holotomographic microscopy analysis using 3D Cell Explorer HM^®^ (Nanolive, Lausanne, Switzerland) equipped with 60× magnification (λ = 520 nm, sample exposure 0.2 mW/mm^2^) and a field depth of 30 µm. All live 3D-holotomographic images were analyzed using STEVE^®^ software (Nanolive, Lausanne, Switzerland) to obtain a refractive index-based Z-stack and digital staining according to different intracellular organelle-based refractive indices (RI).

SEM-based illustrations were performed on *C. parvum*-infected and non-infected HCT-8 cell layers (negative controls) fixed in 2.5% glutaraldehyde (Merck) and post-fixed in 1% osmium tetroxide (Merck). After washing in distilled water and dehydration, samples were dried by CO_2_-treatment to a critical point and sputtered with gold particles. Samples were analyzed using a scanning electron microscope (Philips XL30^®^, Dreieich, Germany) at the Institute of Anatomy and Cell Biology, Justus Liebig University Giessen, Germany, as described by Fichtner et al. [53].

### 2.5. Vicia Villosa Lectin-Based Detection of C. parvum Infections

Cell layers were washed thrice in sterile 1× PBS, fixed in 4% paraformaldehyde (Merck, 15 min) and washed again thrice in 1× PBS. For permeabilization and blocking, host cells were treated with 0.3% Triton X-100 (T-8787, Sigma-Aldrich) and 3% bovine serum albumin (BSA, Sigma-Aldrich) for 1 h, at RT. For parasite detection, biotinylated *Vicia villosa*-derived lectin (B-1235-2, VVL, 1:2000 dilution, vector laboratories) was added (45 min, RT, dark chamber) to the samples. Thereafter, all samples were washed three times in sterile 1× PBS. For visualization of host cell nuclei, DNA was stained by adding 3% BSA-solution containing Hoechst 33258 staining (Sigma-Aldrich, 1 µg/mL; 15 min,). To visualize host cell membranes, cells were additionally stained by anti-β-catenin (13–8400, Invitrogen, 1:500 dilution, 15 min). Thereafter, samples were carefully mounted in anti-fading buffer Fluoromont-GTM (Invitrogen), and allowed to dry (24 h, RT, dark chamber). Samples were analyzed with an epifluorescence microscope (IX81, Olympus, 100× magnification). Two images were obtained with 40× magnification on each technical replicate (*n* = 6) and merged for intracellular parasite identification and quantification of infection rates using ImageJ^®^ [National Institute of Health (NIH), Maryland, MD, USA].

### 2.6. Quantification of Metabolic Conversion Rates in C. parvum-Infected Host Cell Culture Supernatants

HCT-8 were cultured in 24-well plastic tissue culture plates (Greiner; *n* = 9) and infected with *C. parvum* sporozoites. Non-infected HCT-8 cell layers were used as negative controls and equally processed as *C. parvum*-infected ones. Three hours after the infection, the cell culture medium was exchanged (exactly 1 mL/well) and the cells were further cultivated under physioxic (5%) or hyperoxic (21%) conditions as described in the relevant experiments. Infection rates were controlled by VVL-based fluorescence assays. At 24 h p. i., cell culture supernatants were collected and centrifuged (400× *g*, 10 min, 4 °C) to remove cell debris or remnant parasites. Supernatants were collected, aliquoted, immediately frozen in liquid nitrogen and thereafter stored at −80 °C until further metabolic analysis.

Additionally, the same above-mentioned procedure was performed with HCT-8 cells cultured in RPMI 1640 cell culture medium containing very low glutamine concentration (0.02 mM).

For estimation of metabolic conversion rates in glutamine-supplemented (2 mM) and glutamine-starved (0.02 mM) HCT-8 cells, frozen medium samples were heated for 15 min at 80 °C and subsequently centrifuged at 8000× *g* for 10 min. Glucose, pyruvate, lactate, glutamine, glutamate, serine, alanine and aspartate concentrations were determined using a Respons^®^ 920 bench top clinical analyzer (DiaSys Diagnostic Systems GmbH, Holzheim, Germany) as described elsewhere [44,45,54,55,56]. Metabolic conversion rates were calculated in nanomoles per (h × 10^5^ cells) relative to control medium samples which were incubated in parallel in 24-well plastic tissue culture plates (Greiner) as reference.

### 2.7. Host Cell Toxicity Assays

Host cell toxicity of inhibitors was controlled by both XTT^®^ metabolic cell proliferation assay (11465015001, Merck, Darmstadt, Germany) and photometric lactate dehydrogenase (LDH) activity measurements in cell supernatants as a measure of LDH release from host cells. For LDH measurements, HCT-8 cells were pre-cultured in 96-well plates (Greiner) for 80% confluence. Thereafter, the medium was changed and host cells were cultured for 24 h in presence of respective metabolic inhibitors: [galloflavin (14846, Biomol GmbH, Hamburg, Germany): 200, 400, 800 and 1600 µM; lonidamine (14640, Biomol GmbH): 75, 150, 300 and 600 µM, syrosingopine (SML1908, Merck, Darmstadt, Germany) and compound 968 (352010, Merck, Darmstadt, Germany): 2.5, 5, 10, 20, 40, 80, 160 and 320 µM]. In order to simulate the time period of parasite infection, cells were washed thrice and fresh cell growth medium without inhibitor was added for 3 h. Thereafter, fresh inhibitor-supplemented medium was added and host cells were incubated for additional 21 h. Supernatants were collected and centrifuged (400× *g*, 5 min), frozen in liquid nitrogen and stored at −80 °C. LDH activity was measured photometrically according to Bergmeyer 1974 [57], using a Respons^®^ 920 bench top clinical analyser (DiaSys Diagnostic Systems GmbH). For determination of cell viability, host cells were analysed according to the protocols of XTT^®^ (11465015001, Merck, Darmstadt, Germany, Holzheim, Germany) metabolic proliferation assay. Control cells were incubated with growth medium and 0.1% dimethyl sulfoxide (317275, DMSO, Merck, Darmstadt, Germany) since this was the highest vehicle concentration used for treatments.

### 2.8. Inhibition Assays

All inhibitors were solved in dimethyl sulfoxide (DMSO, Sigma, Darmstadt, Germany). Control host cell layers were incubated in growth medium containing the DMSO concentration added in the approach with the highest inhibitor concentration.

HCT-8 were seeded on round glass coverslips (10 mm diameter, Thermo Fisher Scientific, Braunschweig, Germany) previously pre-coated with 40 µg/mL fibronectin (F1141, Merck, Darmstadt, Germany) and placed in 24-well formats. Three technical replicates were used for each experimental condition. Host cells were grown to 80% confluence under physioxic- and hyperoxic conditions. 24 h before infection, respective inhibitors were added in fresh medium to host cell monolayers: galloflavin (400 µM), lonidamine (150 µM), syrosingopine (10 µM) and compound 968 (10 µM). In parallel, non-treated *C. parvum*-infected HCT-8 were co-cultured as infection controls. At 24 h p. i., cells were fixed and analyzed for infection rates using VVL-based fluorescence assays. For the experimental procedure of parasite infection and pharmacological treatments, refer to Supplementary Figure S1.

### 2.9. Analysis of Glycolytic Responses via Seahorse Technology

For analysis of *C. parvum* infection- and inhibitor-induced glycolytic responses, the Glycolysis Stress Test Kit^®^ (Agilent, Rathingen, Germany) was applied using a Seahorse XFp extracellular flux analyser^®^ (Agilent, Rathingen, Germany). Therefore, 10^3^ HCT-8 cells (triplicates for each experimental condition) were seeded on XFp cell microplates^®^ (Agilent, Rathingen, Germany) for 24 h (~80% of cell confluence) and treated mimicking the treatment scheme implemented for the inhibitory assays (see Section 2.3 and Figure S1). For inhibitor-induced glycolytic changes on host cells, the following inhibitor concentrations were used: galloflavin (25, 50, 100, 200, 400 µM), lonidamine (75, 150, 300 µM), syrosingopine (5, 10, 20 µM) and compound 968 (5, 10, 20 µM). Cells were treated and infected following the same treatment/infection scheme as used for inhibition assays (see Section 2.3 and Section 2.8).

Extracellular flux measurements detecting mitochondrial oxygen consumption rates (OCR) and extracellular acidification rates (ECAR) were performed applying a Glycolysis Stress Test Kit^®^ (Agilent, Rathingen, Germany) according to the manufacturer’s instructions to evaluate both the cellular glycolytic status and adequate inhibitor doses. Therefore, Seahorse XF 8-well plastic cartridges^®^ (Agilent, Rathingen, Germany) were hydrated with distilled water and placed in an incubator lacking CO_2_ supply (37 °C, 45 min). Before measurements, cell culture medium of all samples was replaced by Dulbecco’s Modified Eagle Medium (DMEM)-based medium (Agilent, Rathingen, Germany) supplemented with 2 mM glutamine (Sigma-Aldrich, Darmstadt, Germany). Applying the manufacturer’s protocols, quantitative measurements of OCR and ECAR were performed after sequential supplementation of glucose (10 mM), oligomycin (2.9 µM) and 2-DG (60 mM) (supplied by the kit) via instrument-own injection ports. Immediately after measurements, cells were fixed and counted for normalization. Glycolysis-related data obtained from Seahorse XF Wave software^®^ (Waldbronn, Germany) were plotted using GraphPad Prism^®^ 8 software (San Diego, SD, USA).

In addition, to analyze the effect of *C. parvum* on glycolysis and glycolytic ATP production in infected host cells, the Seahorse XFp Glycolytic Rate Assay^®^ (Agilent, Rathingen, Germany) was here used applying the same experimental conditions as described above and adding sequentially Rotenone and Antimycin A (Rot/AA) (0.5 µM) and 2-deoxy-D-glucose (2-DG) (50 mM) (supplied by the kit). In all cases, three replicates were included. By assuming a CO_2_ contribution factor of 0.50 [58] and considering that HCT-8 share classical tumour characteristics by depending on glycolysis rather than on mitochondrial oxidative phosphorylation for energy supply [59,60,61], proton efflux rates (PER) were additionally calculated by means of wave software and plotted using GraphPad Prism^®^ 8 software(San Diego, SD, USA).

### 2.10. Statistical Analysis

Overall, data were expressed as mean ± SD. Evaluation of metabolite conversion rates in supernatants of *C. parvum*-infected and non-infected HCT-8 cells was performed by *t*-test for independent samples applying the module BMDP3D of the statistical program package BMDP/Dynamic, Release 8.1 (Berkeley, USA) [62] on six replicates for each of the four conditions, namely, infected and non-infected under physioxia (5% O_2_) and hyperoxia (21% O_2_).

Likewise, glycolysis and glycolytic ATP production in *C. parvum*-infected HCT-8 as well as parasite replication were evaluated by means of *t*-test using GraphPad Prism^®^ 8 software (San Diego, CA, USA). For evaluation of the inhibitor effect on HCT-8 glycolytic function a one-way (analysis of variance) ANOVA followed by Dunnett’s test were performed after confirming normality on data by means of Shapiro-Wilk test using of GraphPad Prism^®^ 8 software(San Diego, CA, USA).

In general, for valuation statistical significances a statistical significance level of α = 0.05 was used. Results with *p*-values lower or equal 0.05 were assessed as statistically significant.

## 3. Results

### 3.1. C. parvum Infective Stages and Infection Development in HCT-8 Cells Depend on Oxygen Pressure

**Figure 1:**
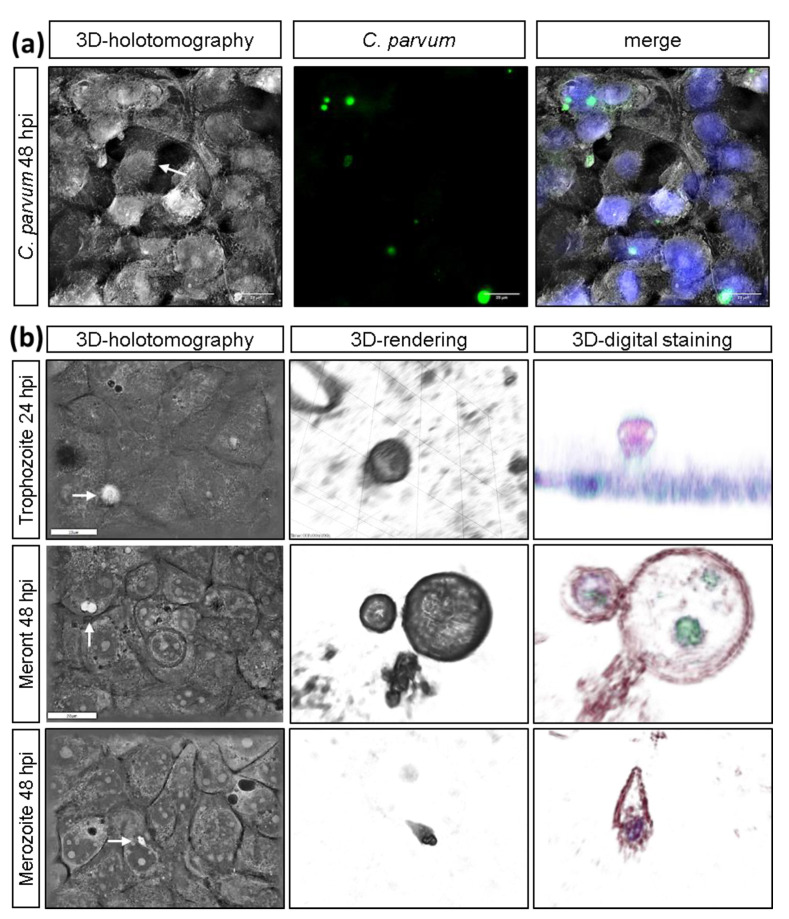
3D-holotomographic illustration of *C. parvum*-infected HCT-8. (**a**) *C. parvum*-infected HCT- 8 cells were stained by biotinylated VVL and Hoechst 33258 at 24 and 48 h p. i. (*n* = 3): (**a**) 3D-holotomographic images were obtained by using 3D Cell Explorer microscope (Nanolive) at 60× magnification (λ = 520 nm, sample exposure 0.2 mW/mm^2^) and a depth of field of 30 µm. (**b**) Holotomography of *C. parvum*-infected HCT-8 and identification of trophozoite, meront and merozoite stages (Scale bar = 20 µm).

Live cell 3D-holotomographic microscopy analysis confirmed parasite-related specificity of VVL-based staining in real time (Figure 1a) and, more importantly, in 3D dimensions. By following intracellular development of sporozoites, we identified different *C. parvum* stages, such as trophozoites and meronts and confirmed their specific epicellular localization in host cells (Figure 1b). The lumen-oriented, intracellular but extracytoplasmatic location of *C. parvum*-derived trophozoite was clearly evidenced in HCT-8 infected-host cells (trophozoite, Figure 1b, 3D-digital staining). The difference in the RI of surface and content from analyzed trophozoites is probably dependent on parasitophorous vacuole (PV)-inherent characteristics. Since the PV is one of *C. parvum*-hallmarks, it is well described that trophozoites always reside inside a PV. The content of here studied trophozoites showed an almost homogenic content, which contrasted with several chambers (up to four, probably meront type II) evidenced in different transversal planes of parasitic stages explored at 48 h p. i. (Figure 1b, second row). At 48 h p. i., a *C. parvum*-merozoite stage was illustrated (Figure 1b, third row) during the attachment and active host cell invasion process. Further, villi-like structures were observed in live infected-host cells (Figure 1a, white arrow).

**Figure 2:**
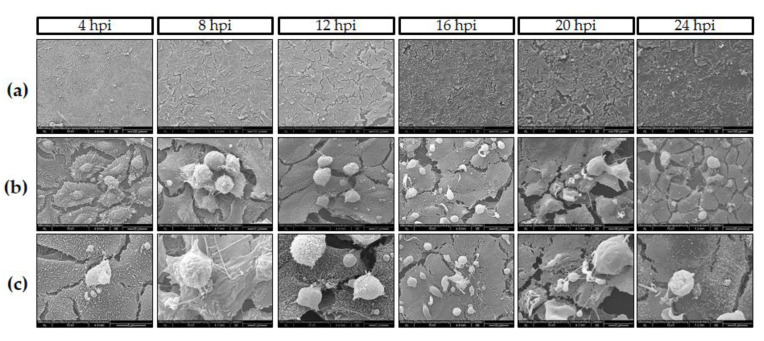
*C. parvum* development in HCT-8 cells under hyperoxic (21% O_2_) conditions (*n* = 6). SEM-based illustration of *C. parvum*-infected HCT-8 evidenced rapid *C. parvum* development and infection kinetics: (**a**) Overviews at different time points post infection (4–24 h p. i.). (**b**,**c**) Closer views of parasite stages-host cell interactions, i.e., column at 16 h p. i. shows meront-infected cells, row b evidenced hole-like damage on host cell surface induced by merozoites release at the same time point (white arrow). Row c (4 h p. i.) reveals early induction of villi-like structures on surface of infected cells (black arrows).

Moreover, “hole”-like cell membrane damages associated with *C. parvum* meront- and/or gamont-driven ruptures of infected host cells were evidenced by SEM analysis, confirming superficial host epithelial cell features in affected villi *in vivo*. SEM analyses visualized gradual *C. parvum* replication resulting in trophozoite-like structures since 4 h p. i. (Figure 2c) and massively observed at 16 h p. i. (Figure 2a). As stated above, also *C. parvum*-infected host cells showed hole-like cell membrane damage after meront rupture, (Figure 2b, 16 h p. i. white arrow), evidencing that some *C. parvum* trophozoites underwent very fast development into mature meront stages and released merozoites in vitro. In addition, villi-like structures were also evidenced by SEM-analysis in *C. parvum*-infected host cells carrying meront stages (Figure 2c, 4 and 12 h p. i. black arrows) as previously reported elsewhere [37].

**Figure 3:**
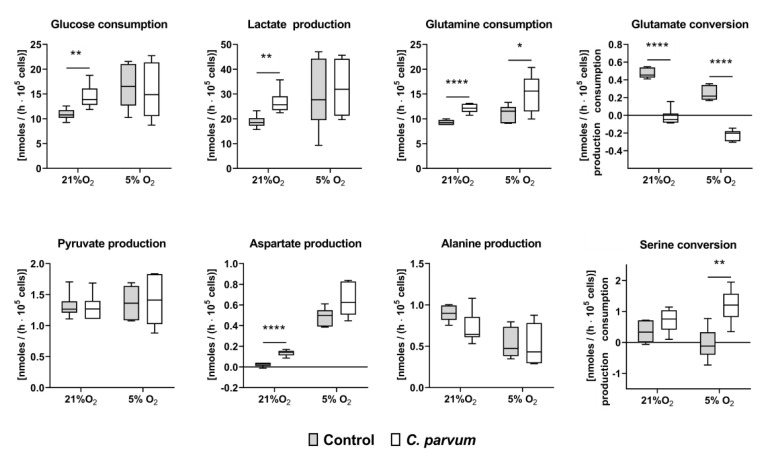
Glucose, lactate, pyruvate, aspartate, glutamine, glutamate, alanine and serine conversion rates in uninfected and *C. parvum*-infected HCT-8 cultivated in glutamine supplemented medium (2 mM) under hyperoxic (21% O_2_) and physioxic (5% O_2_) conditions (*n* = 6, each group). Metabolite conversion rates were analyzed at 24 h p. i. in the cultivation supernatants of the cells and presented as interleaved box and whiskers plots with line at median, bars indicating maximum and minimum values. Statistical significance (* = *p* ≤ 0.05, ** = *p* ≤ 0.01, **** = *p* ≤ 0.0001) was evaluated by *t*-test for independent samples.

**Figure 4:**
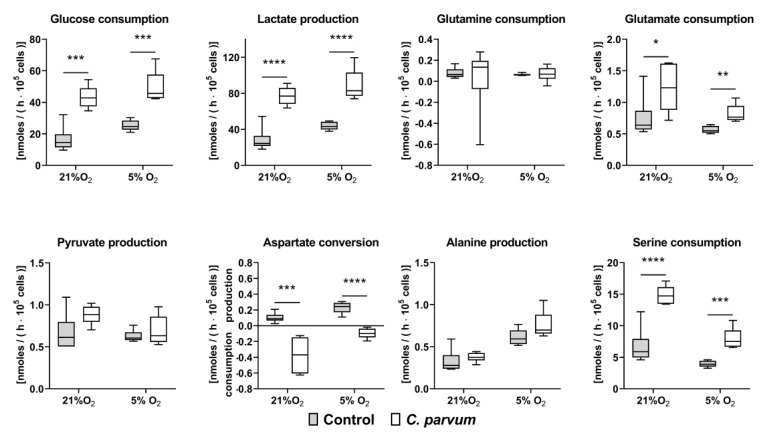
Glucose, lactate, pyruvate, aspartate, glutamine, glutamate, alanine and serine conversion rates in uninfected and *C. parvum*-infected HCT-8 cultivated in glutamine-starved medium (0.02 mM) at hyperoxia (21% O_2_) and physioxia (5% O_2_) (*n* = 6, each group). In comparison to glutamine-supplemented cultivation conditions the parasite infection rate slightly dropped in glutamine starved medium (21% O_2_: 40 ± 3%; 5% O_2_: 28 ± 7%). Conversion rates were analysed at 48 h p. i. in the cell culture supernatants of the cells. Metabolic conversion rates are plotted as interleaved box and whiskers plots showing minimum to maximum values with line at the medians. Statistical significance (* = *p* ≤ 0.05, ** = *p* ≤ 0.01, *** = *p* ≤ 0.001, **** = *p* ≤ 0.0001) was determined by *t*-test for independent samples.

O_2_-dependent *C. parvum* replication in HCT-8 cells under physioxic (5% O_2_) and hyperoxic (21% O_2_) in vitro culture conditions was evaluated in at least three experimental series. When applying parasite stages from identical excystation processes on HCT-8 cells propagated under the above mentioned O_2_ conditions, the lower oxygen disposability (5% O_2_) generally resulted in lower infection rates. In addition, infection rates generally varied with individual excystations. Referring to the different experimental settings, the following infection rates were detected: 46 ± 21% (5% O_2_) and 75 ± 10% (21% O_2_) in experiments on measurement of metabolic conversion rates when cells were cultivated in presence of 2 mM glutamine in the medium (Figure 3) and 28 ± 7% (5% O_2_) and 40 ± 3% (21% O_2_) when cells were cultivated in glutamine-starved medium (0.02 mM glutamine) (Figure 4). 15/45 ± 9/6% (5% O_2_) and 17/53 ± 2/15% (21% O_2_) in repeated experiments on metabolic blockers in the presence of 2 mM glutamine.

### 3.2. Metabolic Signatures of C. parvum-Infected HCT-8 Cells Depend on Oxygen and Glutamine Supply

*C. parvum*-infected and non-infected HCT-8 cells were cultivated and passaged under hyperoxic (21% O_2_) and physioxic (5% O_2_) conditions in the presence of 2 mM glutamine (2 mM glutamine = glutamine-supplemented cells). The metabolic signature of *C. parvum*-infected host cells was characterized by measuring the conversion rates of selected metabolites in the medium supernatants of infected cells comparing to non-infected controls.

When cultured in the presence of 21% O_2_ (hyperoxia) *C. parvum* infection was associated with a significant increase of glucose consumption, lactate production, glutamine consumption and aspartate production as well as a reversal in glutamate conversion from consumption to production (Figure 3). The consumption of glutamine also significantly increased in the infected cells during hypoxia while the difference in lactate and aspartate production was no longer significant when cultivated in presence of 5% O_2_ (physioxia). However, when cultivated in the presence of 5% O_2_, there was a change in serine conversion rates from production in non-infected control cells to consumption in *C. parvum*-infected host cells. During serinolysis, serine is converted to glycerate 2-P and infiltrated into the glycolytic pathway whereby the amino group of serine is transferred to pyruvate with production of alanine. Alternatively, serine can be converted to glycine and cysteine which are precursors for the synthesis of nucleic acid, phospholipids and glutathione. Pyruvate as well as alanine production were not significantly influenced by the infection in either hyperoxia or physioxia (Figure 3). Together these results point out a *C. parvum*-induced increase of intracellular glycolysis and glutaminolysis, independent from oxygen pressure. Glycolysis proved here to play a pivotal role in *C. parvum* replication. By degrading glucose to lactate not only energy is released but cell building blocks are provided to synthesize amino acids, fatty acids and sterols.

Glutaminolysis summarizes the degradation of the amino acid glutamine to pyruvate, CO_2_, lactate and citrate in the course of these recruiting reactions steps of the citrate cycle. The special role of glutamine metabolism in *C. parvum*-infected host cells was also confirmed by metabolic changes which occurred when HCT-8 cells were cultivated under glutamine deficiency conditions (0.02 mM glutamine). Thus, in glutamine-starved *C. parvum*-infected host cells, the glycolytic conversion rates increased more than two-fold as reflected by a highly significant increase of glucose consumption and lactate production at both hyperoxia and physioxia (Figure 4). Furthermore, glutamine deficiency seems to be compensated by an increased uptake of other amino acids. Accordingly, aspartate conversion changed from production to consumption and serine consumption increased in glutamine-starved *C parvum*-infected HTC-8 cells.

Thus, in addition to glucose degradation via glycolysis, the increasingly produced lactate in glutamine supplemented *C. parvum*-infected HCT-8 cells may also derive from degradation of the amino acid glutamine (glutaminolysis).

**Figure 5:**
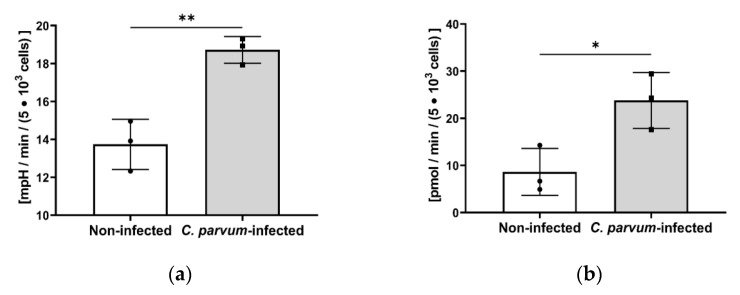
Effect of *C. parvum* infection on glycolysis and glycolytic ATP production in HCT-8 cells under hyperoxic (21% O_2_) conditions. (**a**) *C. parvum* infection-triggered upregulation of glycolysis in HCT-8 cells as evidenced by Seahorse Glycolysis Stress Test Kit. (**b**) Likewise, significant infection-induced upregulation of glycolysis-derived ATP was observed. Bar graph shows mean ± SD, (*n* = 3). *t*-test was use for evaluation of significance (* = *p* ≤ 0.05, ** = *p* ≤ 0.01).

In order to further analyze the role of glycolysis and glycolytic-derived ATP production, we subjected *C. parvum*-infected HCT-8 cells to Seahorse XFp-based analyses on glycolytic responses. Here, glycolytic rate assays revealed an increase of glycolysis in *C. parvum*-infected HCT-8 cells (*p* > 0.005; Figure 5a) when compared to non-infected host cells. Likewise, a significant increase in glycolytic ATP production was observed (*C. parvum*-infected vs. control cells: *p* = 0.0111; Figure 5b) confirming glycolysis’s pivotal role as an energy source for *C. parvum*.

### 3.3. Effects of Selected Metabolic Inhibitors on C. parvum Infection at 5% and 21% O_2_ Conditions

**Figure 6:**
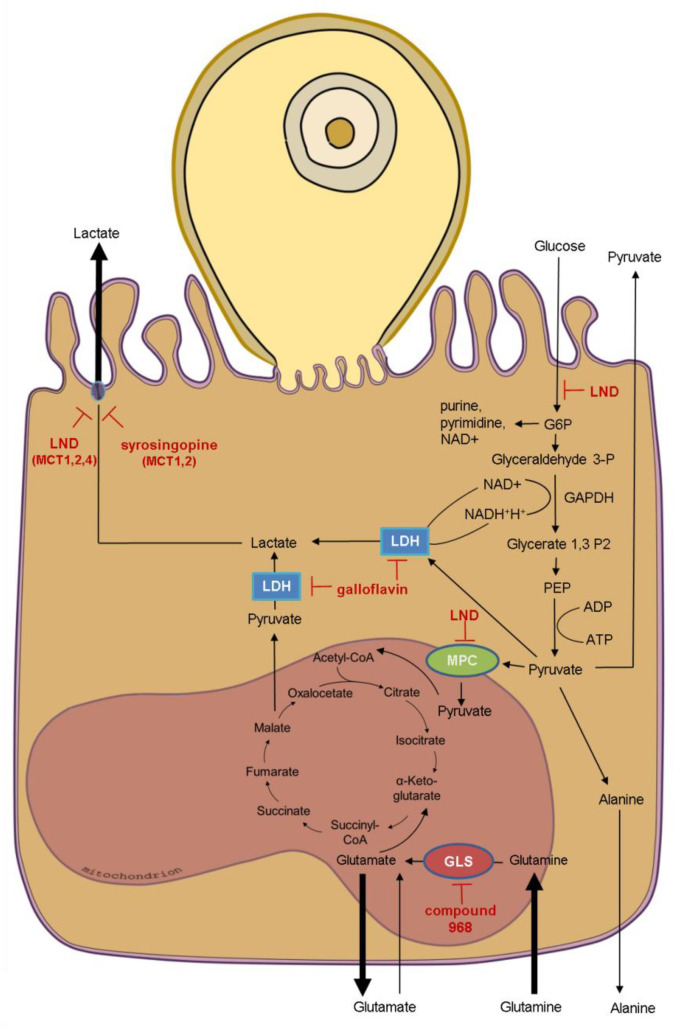
Schematic summary of the metabolic impact of *C. parvum* infection on HCT-8 cells cultivated in presence of 2 mM glutamine and 21% oxygen and targets of selected inhibitors. Bold arrows indicate impact on metabolic conversion rates of *C. parvum*-infected host cells (compare Figure 3). Based on *C. parvum*-induced alterations of host cell metabolism, the indicated metabolic inhibitors were selected to investigate their potential to inhibit *C. parvum* infection in HCT-8 cells. LND: lonidamine, MPC: mitochondrial pyruvate carrier, GLS: glutaminase, LDH: lactate dehydrogenase, MCT: monocarboxylate transporter.

Based on the impact of *C. parvum*-infection on HCT-8 cell metabolism, we examined the influence of the following metabolic inhibitors on *C. parvum* infection rates depending on oxygen supply: galloflavin (inhibitor of lactate dehydrogenase) [63], lonidamine [inhibitor of glycolytic hexokinase (HK), mitochondrial pyruvate carrier (MPC), lactate transporters MCT 1, 2 and 4] as well as succinate dehydrogenase (SD) blocking mitochondrial complex II [64,65,66]. Syrosingopine (inhibitor of MCT1- and MCT4 lactate transporters) [67,68] and compound 968 (inhibitor of glutaminase) [69] (Figure 6).

**Figure 7:**
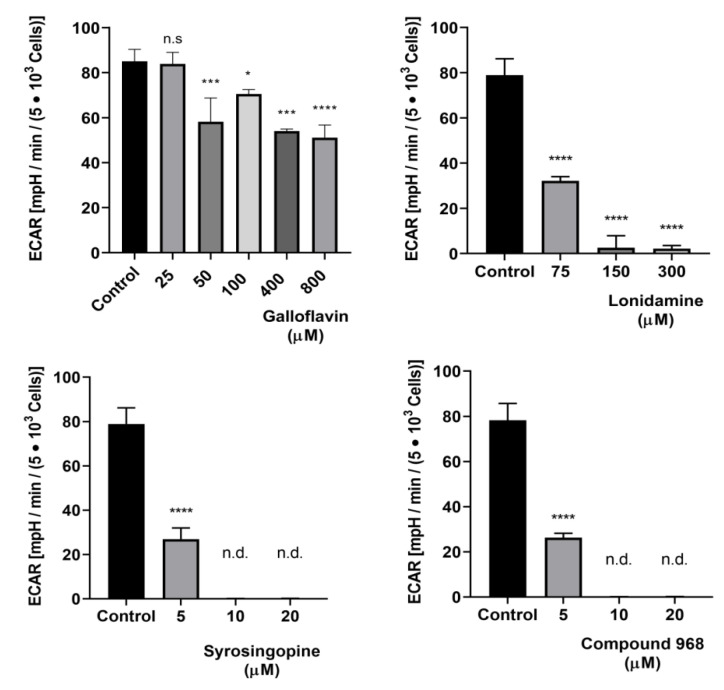
Effects of galloflavin, lonidamine, syrosingopine and compound 968 treatments on glycolysis in uninfected HCT-8 cells cultivated at hyperoxic (21% O_2_) condition. Glycolysis stress test revealed a significant reduction of glycolytic responses by selected inhibitors. Bars represents mean ± SD, (*n* = 3). Data were evaluated for significance by one-way analyses of variance (ANOVA) followed by Dunnett’s test. n.s = non-significant, n.d. = non-detectable, * = *p* ≤ 0.05, *** = *p* ≤ 0.001, **** = *p* ≤ 0.0001.

In preceding dose finding studies, all inhibitors were pretested in uninfected HCT-8 cells for toxicity and glycolysis-lowering effects at different micromolar concentrations (galloflavin: 25, 50, 100, 200, 400, 800, 1600 µM; lonidamine: 75, 150, 300, 600 µM; syrosingopine and compound 968: 5, 10, 20. 40, 80, 160, 320 µM). Overall, there was no evidence of cell toxicity under the treatment ranges used here as detected by both XTT assay and LDH measurements. For controls, cells were mock-treated with 0.1% DMSO (vehicle) representing 100% viability. For positive controls (for cell death), cells were treated with 10% Triton X-100 (Figures S2 and S3). In addition, no alteration of host cell phenotype was visible microscopically at any of the here selected treatment-inhibitor concentration (data not shown). As measured by Glycolysis Stress Test Kit^®^ (Agilent, Rathingen, Germany), all inhibitors led to a significant reduction of glycolysis in uninfected cells, even though to different extents (Figure 7, Figures S5–S8). Of note, compound 968, which represents a potent glutaminase inhibitor, also showed a highly significant glycolysis-blocking efficacy at low micromolar range (10–20 µM, Figure 7).

**Figure 8:**
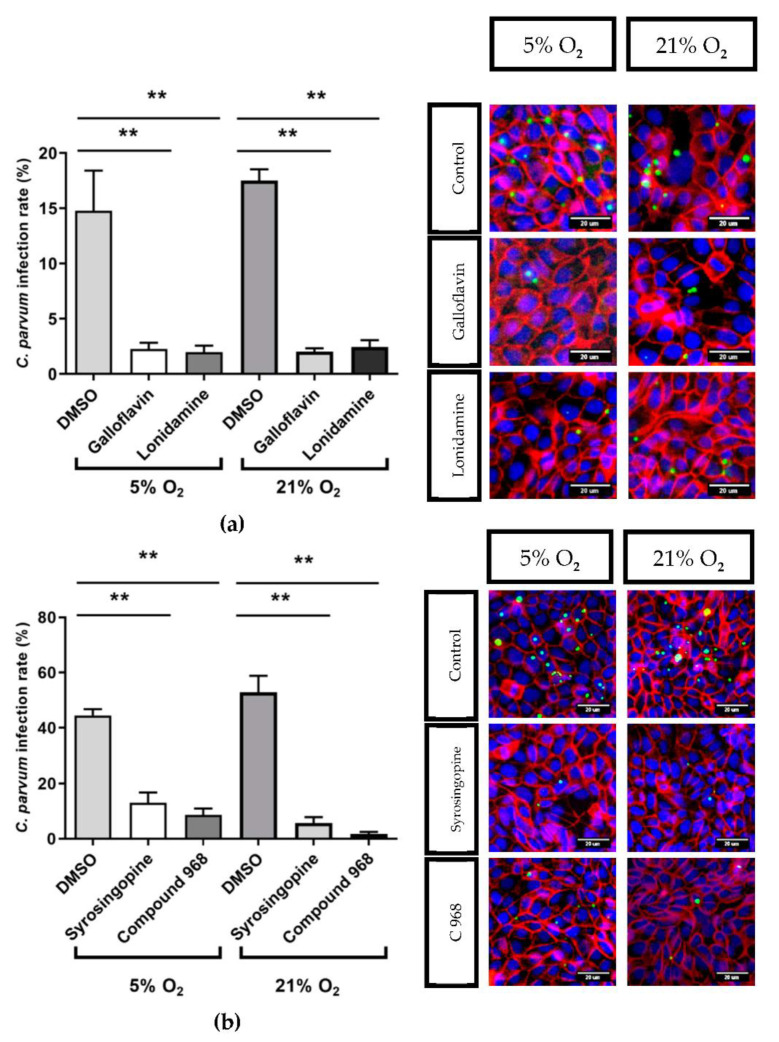
Effects of galloflavin (400 µM), lonidamine (150 µM) (**a**), syrosingopine (10 µM) and compound 968 (10 µM) (**a**,**b**) treatments on *C. parvum* infection rates in HCT-8 cells cultivated at 21% O_2_ and 5% O_2_, and exemplary illustrations of respective inhibition assays. *C. parvum* was stained with VVL, green; host -cell membranes and nuclei were labelled with anti-ß-catenin (red) and Hoechst (blue), respectively. Bars show means ± SD, (*n* = 6). For evaluation of significance *t*-test was performed. ** = *p* ≤ 0.01.

This observation can be explained by the fact that in infected and uninfected HCT-8 cells glucose consumption increased with increasing glutamine consumption which means that glutaminolytic and glycolytic conversion rates positively influence each other (Figure S4) [32]. Based on these preliminary results, the following inhibitor concentrations were chosen to analyse their effects on *C. parvum* infection under physioxic and hyperoxic in vitro conditions: 400 µM galloflavin, 150 µM lonidamine, 10 µM syrosingopine and 10 µM compound 968. The corresponding experimental protocol is described in Supplementary data 1. Overall, treatments with all compounds led to a significant reduction of *C. parvum* infection independent from current O_2_ conditions (galloflavin and lonidamine at both 21% and O_2_ 5% O_2_: *p* < 0.0022, syrosingopine and compound 968 at both O_2_ conditions: *p* < 0.0022). Thus, galloflavin, lonidamine, syrosingopine and compound 968 treatments induced a reduction of intracellular *C. parvum* infection of 80%/88%, 90%/81%, 71%/89% and 81%/97% under 5%/21% O_2_ conditions, respectively (Figure 8).

**Figure 9:**
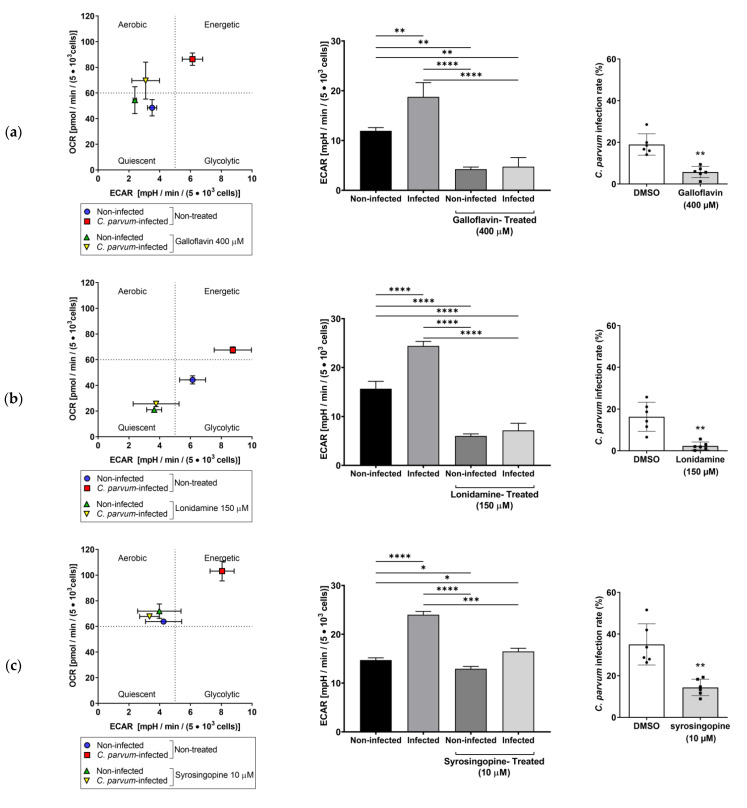

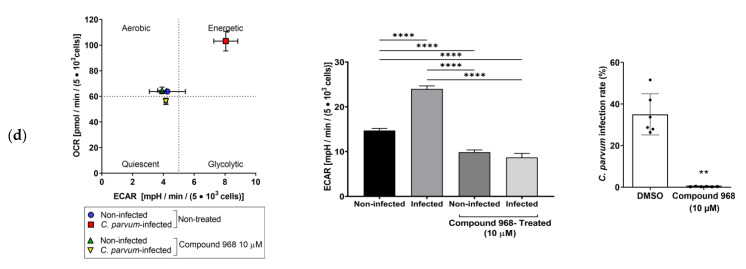
Impact of *C. parvum* infection and inhibitor treatments on HCT-8 energetic profiles: (First column) The current energetic profiles are plotted by presenting OCR- and ECAR values in HCT-8 (*n* = 3, each condition). (Second column) Effect of galloflavin (**a**), lonidamine (**b**), syrosingopine (**c**) and compound 968 (**d**) treatments on energetic profiles of HCT-8 infected and non-infected controls: The glycolytic function was evaluated in *C. parvum*-infected host cells under 21% O_2_ by means of Seahorse Glycolysis Stress Test. Glycolytic activities in treated and infected cells were compared with untreated and infected cells, revealing a significant reduction in glycolytic capacity. (Third column) Thus, reduced glycolytic capacity corresponds with significant reduction of infection rates. Bars present means ± SD, (Energy maps, First column, *n* = 3; Glycolysis, second column *n* = 3, Infection rates, third column, *n* = 6). For evaluation of significance on glycolytic function (second column) one-way analyses of variance (ANOVA) followed by Dunnett’s test was performed. Significances of inhibitors effects on infection rates (third column) were estimated by means of *t*-test. * = *p* ≤ 0.05, ** = *p* ≤ 0.01, *** = *p* ≤ 0.001, **** = *p* ≤ 0.0001.

To further analyze the impact of the different metabolic inhibitors on the energetic status of *C. parvum*-infected host cells, extracellular flux analyses were performed applying Glycolysis Stress Test^®^ (Agilent, Rathingen, Germany). Therefore, *C. parvum*-infected HCT-8 and non-infected controls were subjected to measurements of ECAR and OCR by Seahorse instrumentation and related data were plotted as energetic profiles thereby correlating both measurements and showing a general overview of host cellular energetic status under different treatments (Figure 9 first column).

The related test protocol includes initial measurements of basal non-glycolytic acidification, followed by reactions upon sequential supplementation of glucose (induces glycolysis), oligomycin (blocks ATP synthase) and 2-deoxy-D-glucose (blocks glycolysis) (for back-calculation of glycolytic reserve) via instrument-own injection ports. Energetic maps (Figure 9, first column) revealed that *C. parvum* infection was generally linked to a shift towards a high energetic status and likewise to a rise of glycolysis (Figure 9, second column) when compared to non-infected control cells. When inhibitors were applied, the high energetic status of cells changed and moved back towards the lower energetic status of non-infected control cells. The overall energetic behaviour of inhibitor-treated cells was mirrored by decreased ECAR values. Inhibitor-induced decreases in glycolytic activity was also related with a significant reduction in *C. parvum* infection rates (*p* = 0.0022 for all treatments) (third column of Figure 9). Related data on the glycolytic capacity, glycolytic reserve and non-glycolytic acidification are presented in supplementary files (Figures S5–S8).

## 4. Discussion

Here we analyzed the effect of *C. parvum* on the metabolic signatures of infected HCT-8 under physioxia (5% O_2_) to mimic in vivo physiological gut conditions and found that all, glycolysis, glutaminolysis and degradation of the amino acid serine seemed essential for parasite replication. Whilst *C. parvum* has own glycolytic capacities, it lacks the metabolic equipment for glutaminolysis rendering the latter as pure host cell-supplied activity. Glutaminolysis recruits reaction steps of the citrate cycle which is not present in *C. parvum*. The significant impact of metabolic inhibitors targeting glutamine consumption and lactate release on *C. parvum* infection confirmed the high relevance of both energetic pathways for *C. parvum* replication. Moreover, novel live cell 3D-holotomographic microscopy was performed to unveil parasite-host cell interactions, precise localization of sporozoites and intracellular, trophozoite, meront and gamont stages.

Due to *C. parvum*-minimal own metabolic capacities which are mainly limited to glycolysis, the parasite must modulate host cell metabolism for its intracellular replication [14,15,16,18,28]. In line with these restricted metabolic capacities, phylogenetic findings advocate that ancestral apicomplexan protozoa originally used myzocytosis, a predatory behaviour, by piercing host cell membranes in order to suck nutrients from the host [15,19,36]. Interestingly, *C. parvum* resides intracellularly with an epicellular location forming a feeder organelle, which is in contact with the host cell cytoplasm. This feeder organelle can be seen as a pleisiomorphic feature of such ancestral predatory behaviour, which ensures nutrient uptake from host cells and is now referred to as “cell vampirism” [20,36,69,70,71]. We also detected feeder organelles in early *C. parvum* trophozoite stages by means of live cell 3D-holotomography microscopy, pointing out cell vampirism as an important strategy for successful nutritional uptake.

In the current study we found significant differences in metabolic conversion rates between *C. parvum*-infected and non-infected host cells. A striking example is the increased lactate production rate, which was detected by two independent methods, i.e., by photometric lactate measurements and by determining acidification of *C. parvum*-infected cell-derived supernatants under both physioxia and hyperoxia. Our results confirm recent findings on *C. parvum*- and *C. hominis*-infected mice models [26]. Likewise, D-lactate-derived acidosis has also been described in neonatal calves experiencing clinical cryptosporidiosis [72,73]. Lactate is a final product of glucose degradation in differentiated cells under hypoxic conditions and in proliferating cells under both 21% oxygen and hypoxia. In line, we found significant enhanced glucose consumption rates in *C. parvum*-infected HCT-8 cells when cultured in the presence of 21% O_2_. Likewise, Seahorse-based analyses indicated a significant up-regulation of glycolysis and glycolytic ATP synthesis and showed significantly enhanced ECAR values in *C. parvum*-infected host cells. Our findings in principle are in agreement with previously described *C. parvum*-induced metabolic changes in host cell cultures [26], in *C. hominis*- and *C. parvum*-infected animals [25,26] and with data on transcription control of glycolysis-related genes in *C. parvum* infected HCT-8 cells [23].

Of note, in addition to glucose, amino acids, such as glutamine and serine, can also be degraded to lactate. In *C. parvum*-infected HCT-8 cells, glutamine consumption significantly increased at both hyperoxia and physioxia. In addition, serine conversion was reversed from production in control cells to consumption in *C. parvum*-infected cells at physioxia. The parallel change in glutamate conversion from consumption to production in *C. parvum*-infected HCT-8 at hyperoxia and physioxia indicates that a certain proportion of consumed glutamine was not introduced into citric acid cycle but instead released as glutamate thereby contributing to the acidification of the medium. The crucial role of glutamine metabolism in *C. parvum*-infected host cells was confirmed by metabolic changes which occurred at glutamine-starved in vitro culture conditions (0.02 mM glutamine). In glutamine-starved *C. parvum*-infected host cells, the glycolytic conversion rates increased more than two-fold at both hyperoxia and physioxia as reflected by a highly significant increase in glucose consumption and lactate production. Accordingly, in glutamine-supplemented medium (2.0 mM glutamine) significantly increased glutamine consumption and glutamate production rates were found in *C. parvum*-infected host cells under hyperoxia, as well as under physioxia.

Together, these metabolic findings evidenced a key role of glutaminolysis in parasitized host cells as also described for other apicomplexan parasites, such as *T. gondii* and *Besnoitia besnoiti* [31,32,33]. In the blood of mammalian species, glutamine is an important vehicle of nitrogen, i.e., for nitrogen transport from muscles to liver. Thus, glutamine represents the amino acid with the highest concentration in the blood in mammalians. For *C. parvum* development, glutamine appears to be essential. Glutamine is an important substrate in purine and pyrimidine synthesis. Additional functions of glutamine rely on its conversion to glutamate, which is a precursor for the synthesis of different amino acids and glutathione, which is also used to fuel the TCA cycle. Accordingly, besides glycolysis, glutaminolysis is another important energy source in fast proliferating cells, such as intestinal epithelial cells and tumor cells [43,44,68,74,75,76]. Of note, dynamic cell type-specific interactions between glucose and glutamine metabolism are described in several non-tumor and tumor cells [54,76,77]. This interplay was also the case for the HCT-8 cell line used in this study. In future experiments, we would like to extend the current metabolic characterization to primary intestinal epithelial cells to simulate human and animal *C. parvum*-infections in more realistic cell culture systems. Consistently, there are several studies reporting a key role of glutamine in intestinal epithelial cell proliferation [78,79,80], epithelial cell tight junction integrity [81,82] and in local gut-driven host innate immune reactions [83,84]. In addition, there is also some evidence on glutamine acting as amino acid for maintenance of intestinal barrier function in children [85,86], which are actually the most vulnerable age group of human cryptosporidiosis infections in developing countries worldwide [4,5,6]. The same holds true for lactate, which induces fast and strong activation of mammalian polymorphonuclear neutrophils (PMN) resulting in the formation of neutrophil extracellular traps (NETs) [87,88,89] which represents an essential innate effector mechanism against various invasive pathogens [90], including protozoan and helminth parasites [90]. In line, *C. parvum*-triggered NET formation was also reported to occur in PMN of human and bovine origin [91].

Glutamine has also been proven to decrease intestinal permeability and bacterial translocation in mice and in critical cryptosporidiosis patients [92,93,94]. In line with our present results, indicating a pivotal role of glutamine for *C. parvum* replication, treatments with compound 968, which blocks glutamine degradation by inhibiting mitochondrial glutaminase, led to a highly significant reduction of *C. parvum* development. Compound 968 is a tetrahydrobenzo[a] derivative capable of blocking Rho GTPase-induced transformation in fibroblasts and inhibiting cancer cell growth [69]. Interestingly, after achieving reversion of compound 968 by adding α-ketoglutarate (α-KG) it became clear that fuelling of TCA cycle through glutaminolysis was critical for tumour growth [69,95]. Similar to glucose, the importance of glutamine for cell growth lies both in regeneration of energy and in the supply of starting molecules (here carbon atoms and nitrogen) for biosynthesis of cell building blocks. In fact, a high demand of cell building blocks is present in rapidly dividing cells, such as intestinal epithelial cells, which show a high cellular turnover [78,79,96,97]. Interestingly, compound 968 did also inhibit glycolysis in *C. parvum*-infected HCT-8 cells as reflected by a downregulation of extracellular acidification rate. In non-infected and *C. parvum*-infected HCT-8 cells, an increase in glutamine concentration correlated with an increase in glucose consumption. An explanation for this correlation is that glutamate, the product of the glutaminase reactions, provides the amino group for serine synthesis from the glycolytic intermediate glycerate 3-P. Indeed, serine consumption from culture medium significantly increased in glutamine-starved *C. parvum*-infected cells, presumably due to an impairment of intracellular serine synthesis. Serine is a precursor for the synthesis of cysteine as well as glycine and N5N10 methylene tetrahydrofolate. The latter two are substrates for the synthesis of nucleic acids. [33,74,98]. Cysteine and glycine are necessary for the synthesis of glutathione which plays an important role in detoxification of ROS. Of note, high ROS concentrations are known to impair parasite viability and represent a pivotal mechanism of early host innate immune reactions directed against protozoan and metazoan parasites [99,100].

To address the role of both lactate and the glucose-lactate-axis in physioxic and hyperoxic *C. parvum*-infected HCT-8 cells, we additionally investigated the impact of galloflavin, lonidamine and syrosingopine on infection rates and on the metabolism of HCT-8 cells. It is important to consider that glycolysis-related inhibitors will not exclusively target metabolic pathways of host cells but also of *C. parvum*-driven glycolysis [18,29,60]. Lactate is produced by lactate dehydrogenase-catalyzed reduction of pyruvate thereby regenerating cytosolic NAD+ which is the substrate of the glycolytic GAPDH reaction. Thus, NAD+ deficiency leads to an inhibition of glycolysis. Galloflavin inhibits the lactate dehydrogenase. In accordance to other studies investigating the impact of lactate dehydrogenase inhibitors on *Cryptosporidium* replication in presence of 21% oxygen [27,29,30], galloflavin treatments significantly blocked *C. parvum* infection rates under both oxygen conditions, thereby underlining the crucial role of lactate synthesis even in conditions of physioxia. The increased amount of lactate released by *C. parvum*-infected host cells may derive from an enhanced breakdown of glucose (glycolysis) or of the amino acids glutamine (glutaminolysis) and serine (serinolysis).

In order to avoid an acidification of the cytoplasm, lactate is exported from cells via monocarboxylate transporters (MCTs). Accordingly, we investigated the impact of two different MCT inhibitors on *C. parvum*-infected HCT-8 cells: syrosingopine, which inhibits MCT 1 and MCT2 [67,68] and lonidamine, which is a MCT1-, 2, and 4 inhibitor [66]. As symporters, MCT 1–4 transport lactate and protons into the extracellular space, thus contributing to pH regulation especially during glycolysis [66,101]. MCT inhibition prevents the release of lactate regardless of the metabolic origin (i.e., glycolysis, glutaminolysis or serinolysis). In contrast to syrosingopine, lonidamine induces—besides MCT blockage—also an inhibition of hexokinase (=enzyme in glycolysis), succinate dehydrogenase (=enzyme in citric acid cycle) and mitochondrial complex II (=component of OXPHOS) [64,65]. Both syrosingopine- and lonidamine-induced significant inhibition of *C. parvum* infection in HCT-8 cells independent from the oxygen pressure (hyperoxia or physioxia). Interestingly, in the case of lonidamine, which in addition to MCTs also targets hexokinase, succinate dehydrogenase and complex II, a 15-fold higher concentration had to be used in order to obtain the same level of inhibition of *C. parvum* infection as the obtained by using syrosingopine.

The exact molecular mechanisms on how *C. parvum* sporozoites attach and penetrate intestinal mucus is still not fully understood [15,36]. Effective blockage of active *C. parvum*-sporozoite intestinal mucus migration could constitute an important cornerstone for drug development, since this event is critical for the establishment of infection. Thus, the possibility to follow *C. parvum* development in live cells and real time enables us to better understand such fundamental events. Accordingly, the label-free RI-based digital staining has successfully been used to unveil the intracellular architecture and parasite-host cell interactions of related apicomplexans [102,103] and, more recently, cytopathogenic effects of viruses [104]. In this sense, we here presented first live cell 3D-holotomographic microscopy analyses of *C. parvum* infection. The, refractive index- and nanoscale membrane fluctuation-based analyses revealed feasible and non-invasive approaches to study sporozoite host cell invasion and early sporozoite/host cell interactions [15,102,103].

## 5. Conclusions

Metabolic profiling of *C. parvum*-infected cells evidenced here a parasite dependent-upregulation of glycolysis. The low ATP production efficiency of glycolysis compared to that obtained by oxidative phosphorylation clearly points out a demand of macromolecules and reductive agents necessary for *C. parvum* rapid replication. Interestingly, the correlation found here in glycolysis and glutaminolysis under physiologic O_2_ conditions agree with previous postulates on other *C. parvum*-related parasites (e.g., *T. gondii*), provoking theorization about an evolutionary convergence of apicomplexans and cancer cells. Likewise, we confirm here a *C. parvum* dependence on glutaminolysis, rendering the least one as potential anti-cryptosporidial target.

Moreover, we here showed the usefulness of live cell 3D-holotomographic microscopy for further detailed illustrations, such as the formation of lipid droplets, feeder organelle, parasitophorous vacuole (PV) changes and/or mitochondrial movements in *C. parvum*-infected host epithelial cells. This technique, combined with SEM and transmission electron microscopy (TEM) will help to better understand the dynamic and complex parasite-host cell interactions, such as formation of feeder organelle and myzocytosis, but also allow to precisely evaluate possible impacts of novel anti-cryptosporidial drugs on *C. parvum* intracellular replication.

## Supplementary Materials

The following are available online at https://www.mdpi.com/2079-7737/10/1/60/s1, Figure S1: Treatment/infection scheme. Figure S2: Viability assays of host-cells treated with selected inhibitors by means of XTT. Figure S3: Viability assays of host-cells treated with selected inhibitors by means of LDH measurement. Figure S4: Correlation between glutamine consumption and glucose consumption in *C. parvum*-infected HCT-8 cells under 5% oxygen condition. Figures S5–S8: Glycolytic capacity (a), glycolytic, reserve (b) and non-glycolytic acidification (c) of *C. parvum*-infected-HCT-8 under 21% O_2_ and relative effects of galloflavin (Figure S5), lonidamine (Figure S6), syrosingopine (Figure S7) and compound 968 (Figure S8) treatments.
