# Supplementary material for: Metabolic Signatures of Cryptosporidium parvum-Infected HCT-8 Cells and Impact of Selected Metabolic Inhibitors on C. parvum Infection under Physioxia and Hyperoxia
Source: Biology (Basel). 2021 Jan 15;10(1):60. doi: 10.3390/biology10010060 (PMC7831031; doi:10.3390/biology10010060)
Supplement: Supplementary file 1 [file biology-10-00060-s001.zip › biology-1009629-supplementary.pptx]

## Slide 1
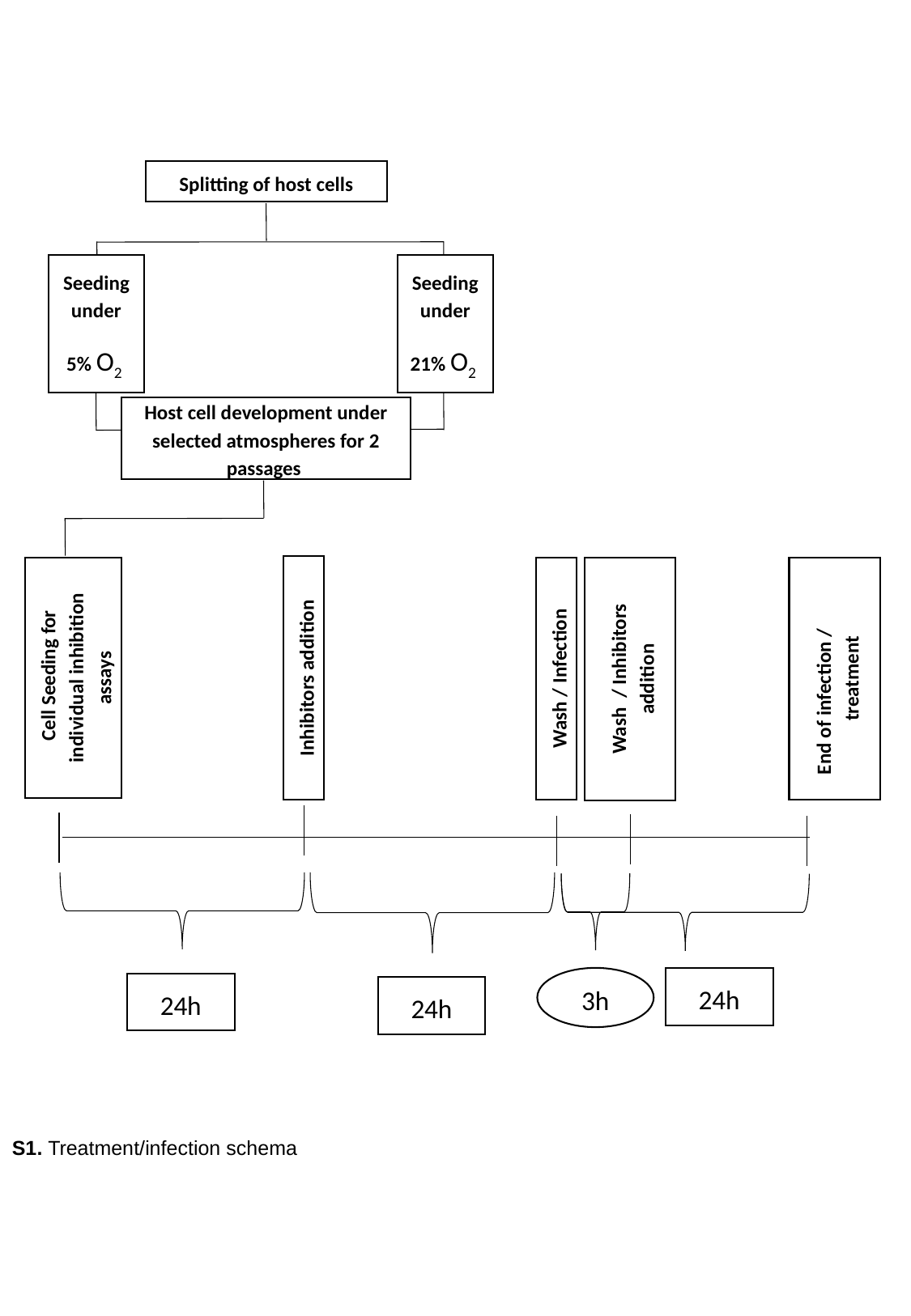

Splitting of host cells
Seeding under
5% O2
Seeding under
21% O2
Host cell development under selected atmospheres for 2 passages
 Cell Seeding for individual inhibition assays
End of infection / treatment
Wash / Inhibitors addition
Inhibitors addition
Wash / Infection
3h
24h
24h
24h
S1. Treatment/infection schema

## Slide 2
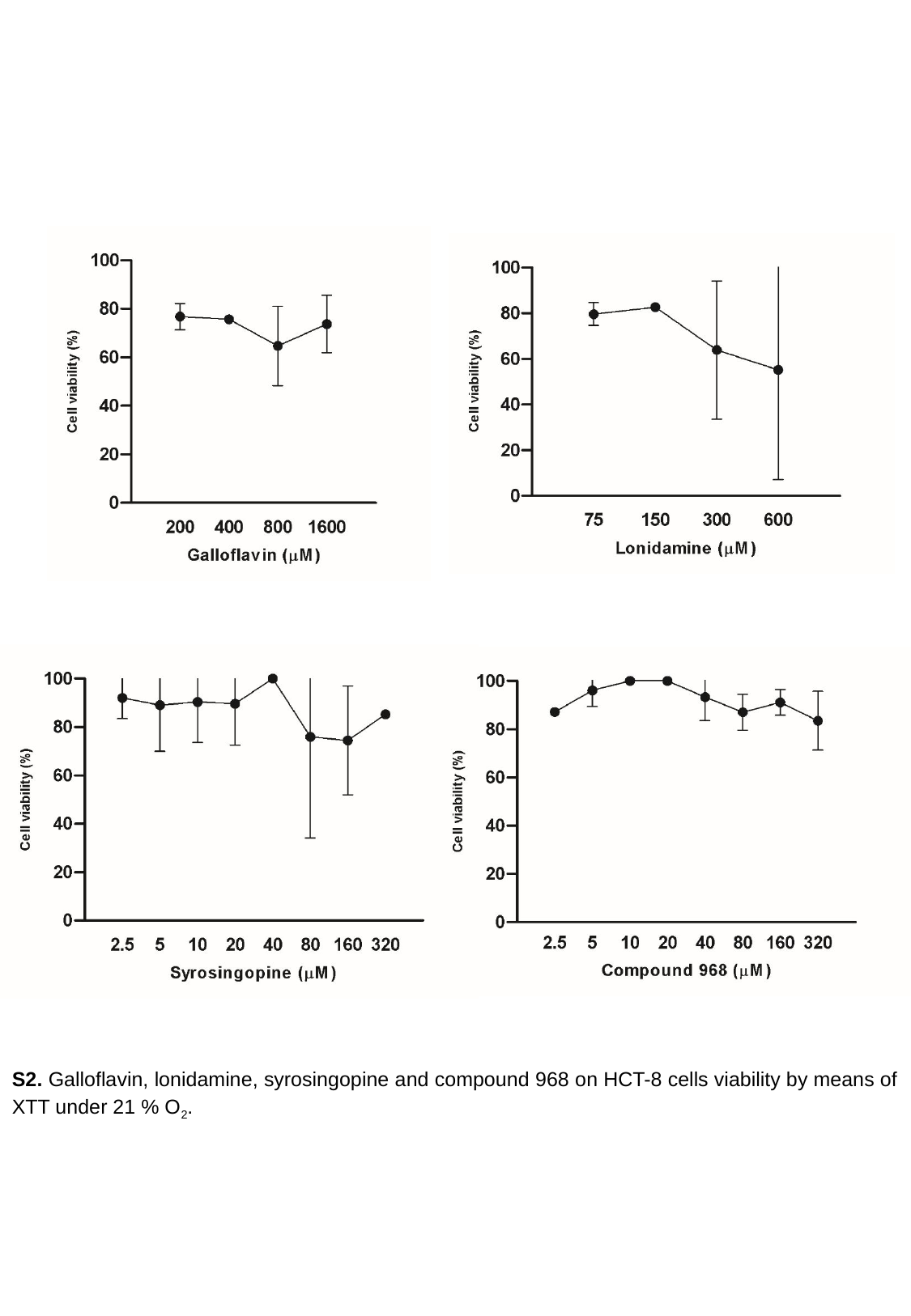

S2. Galloflavin, lonidamine, syrosingopine and compound 968 on HCT-8 cells viability by means of XTT under 21 % O2.

## Slide 3
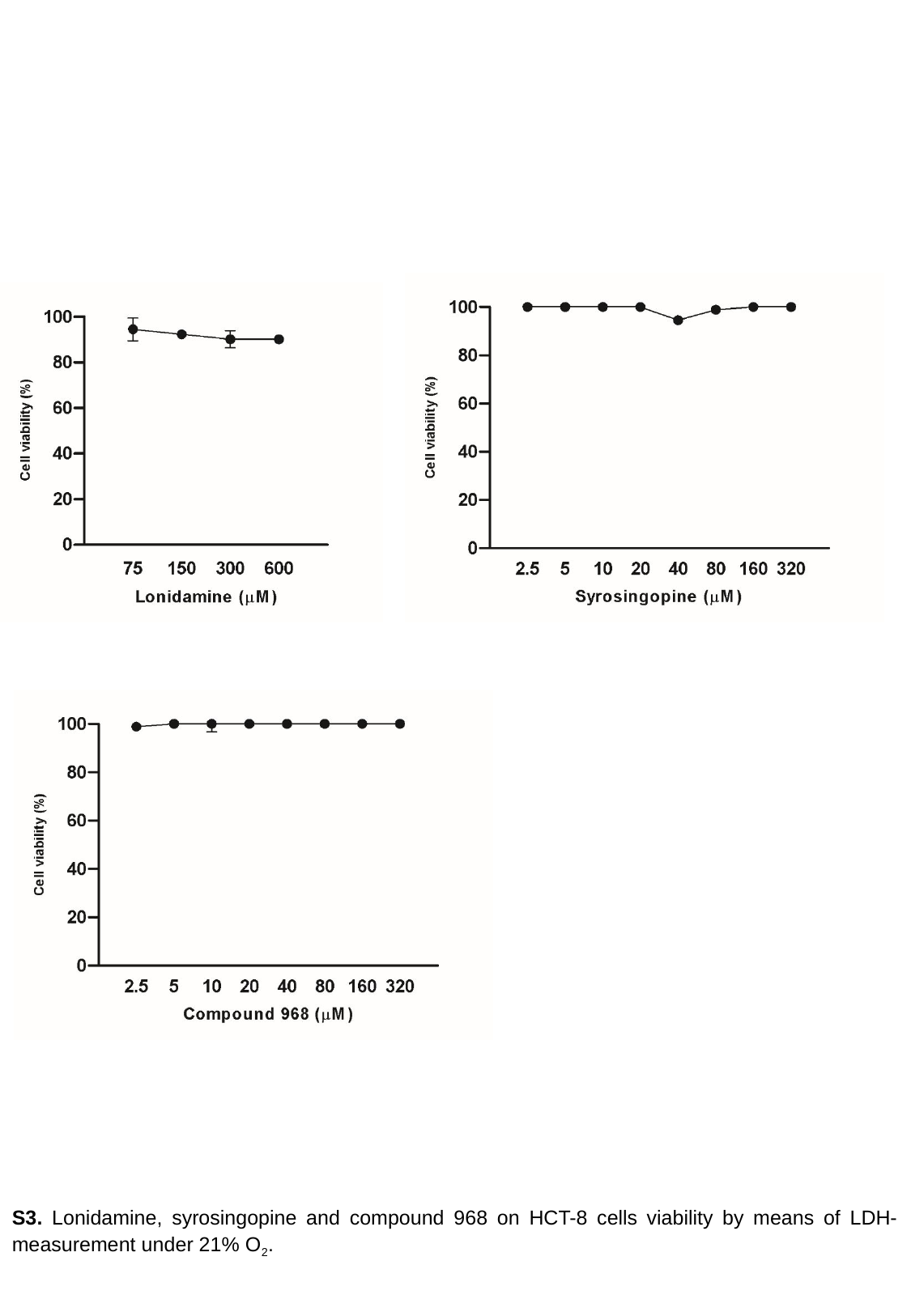

S3. Lonidamine, syrosingopine and compound 968 on HCT-8 cells viability by means of LDH-measurement under 21% O2.

## Slide 4
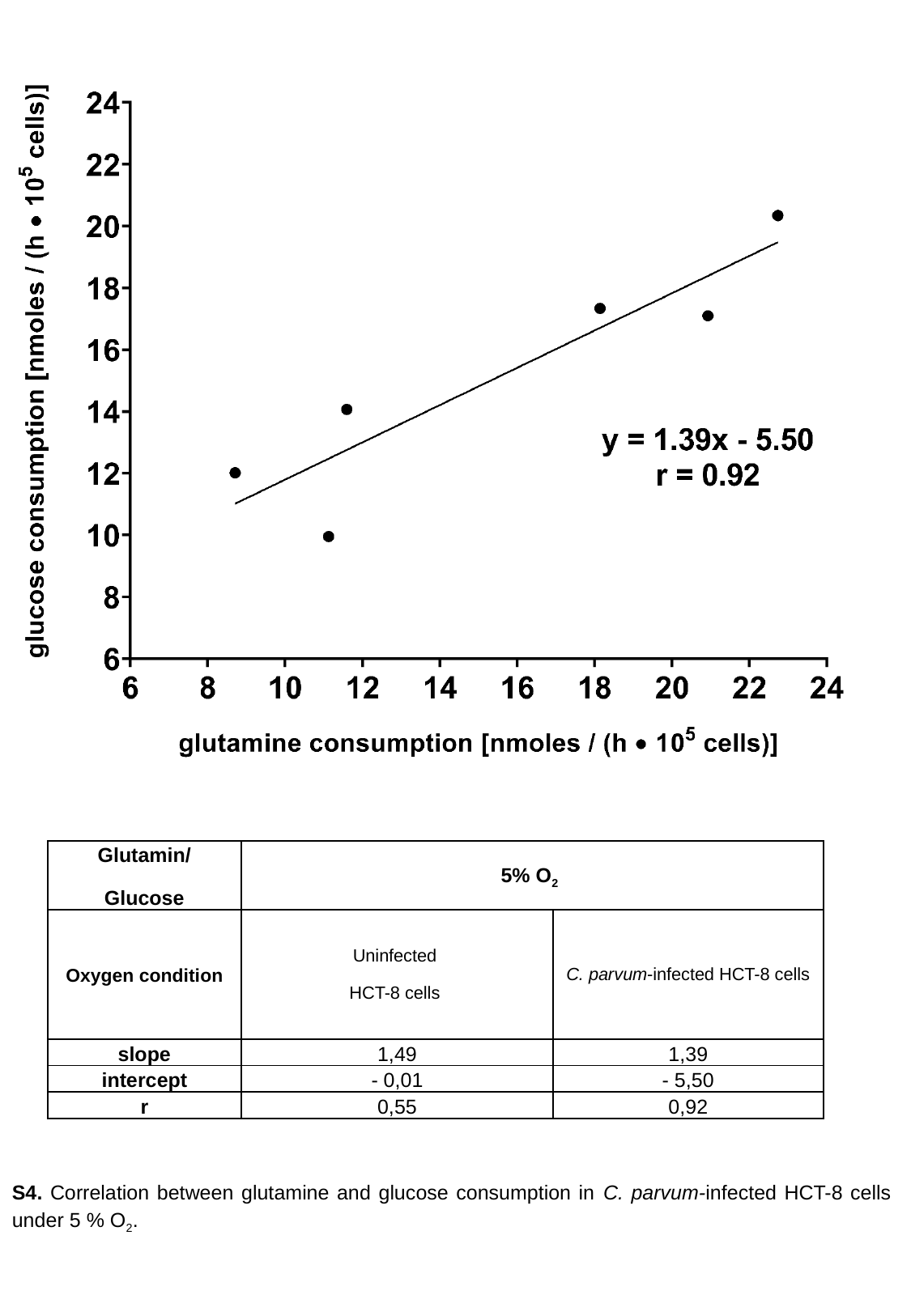

| Glutamin/ Glucose | 5% O2 | |
| --- | --- | --- |
| Oxygen condition | Uninfected HCT-8 cells | C. parvum-infected HCT-8 cells |
| slope | 1,49 | 1,39 |
| intercept | - 0,01 | - 5,50 |
| r | 0,55 | 0,92 |
S4. Correlation between glutamine and glucose consumption in C. parvum-infected HCT-8 cells under 5 % O2.

## Slide 5
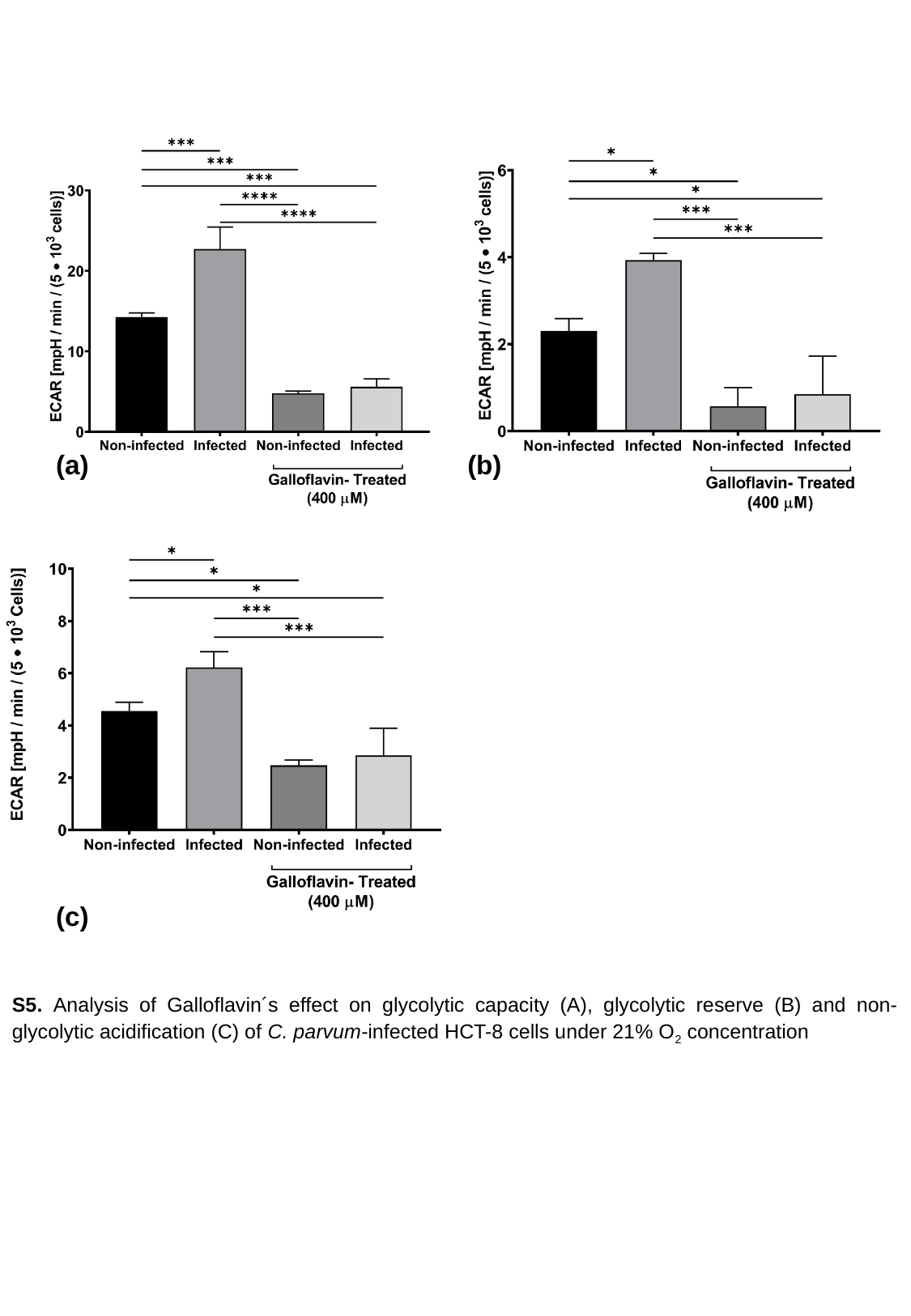

(a)
(b)
(c)
S5. Analysis of Galloflavin´s effect on glycolytic capacity (A), glycolytic reserve (B) and non-glycolytic acidification (C) of C. parvum-infected HCT-8 cells under 21% O2 concentration

## Slide 6
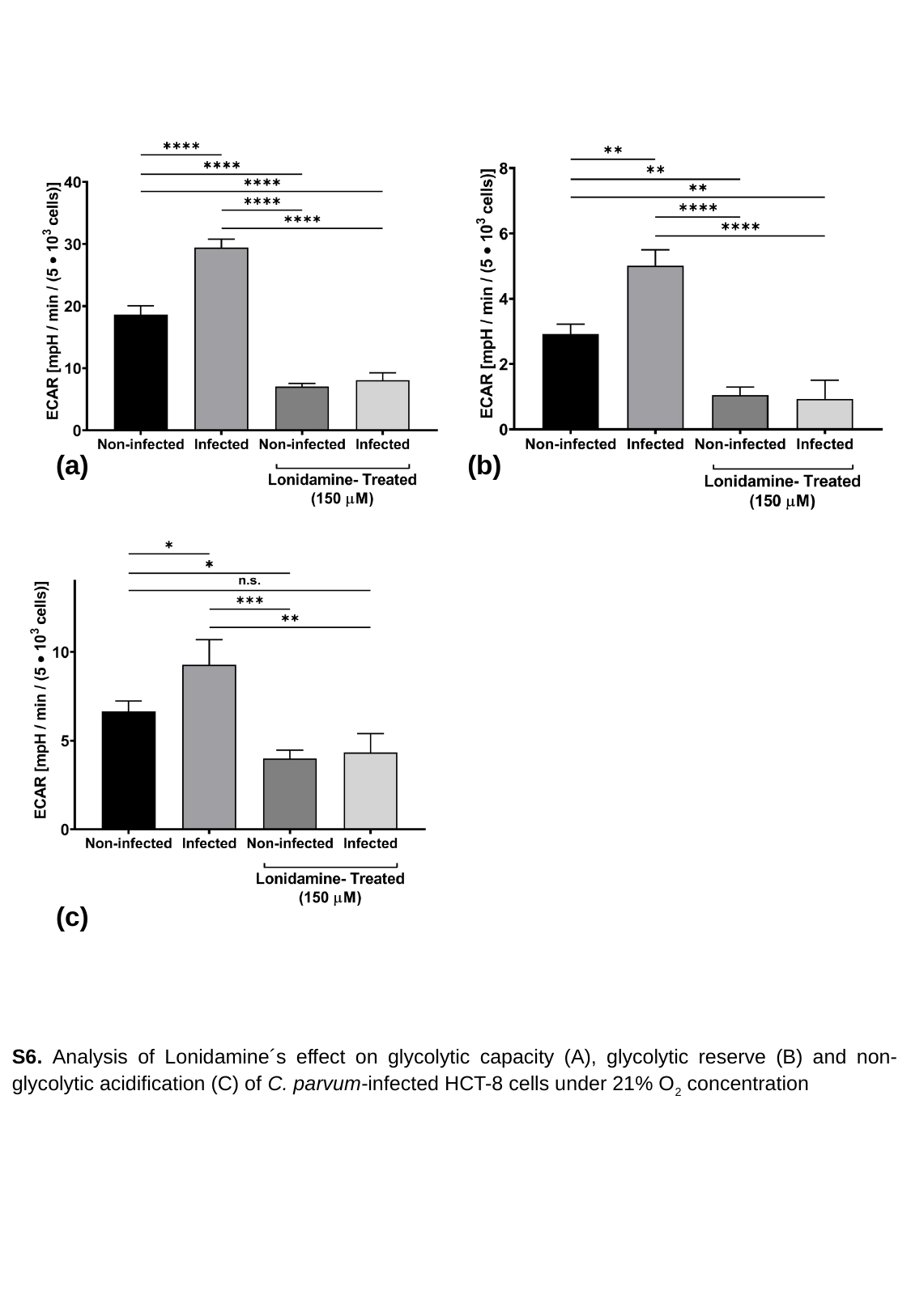

(a)
(b)
(c)
S6. Analysis of Lonidamine´s effect on glycolytic capacity (A), glycolytic reserve (B) and non-glycolytic acidification (C) of C. parvum-infected HCT-8 cells under 21% O2 concentration

## Slide 7
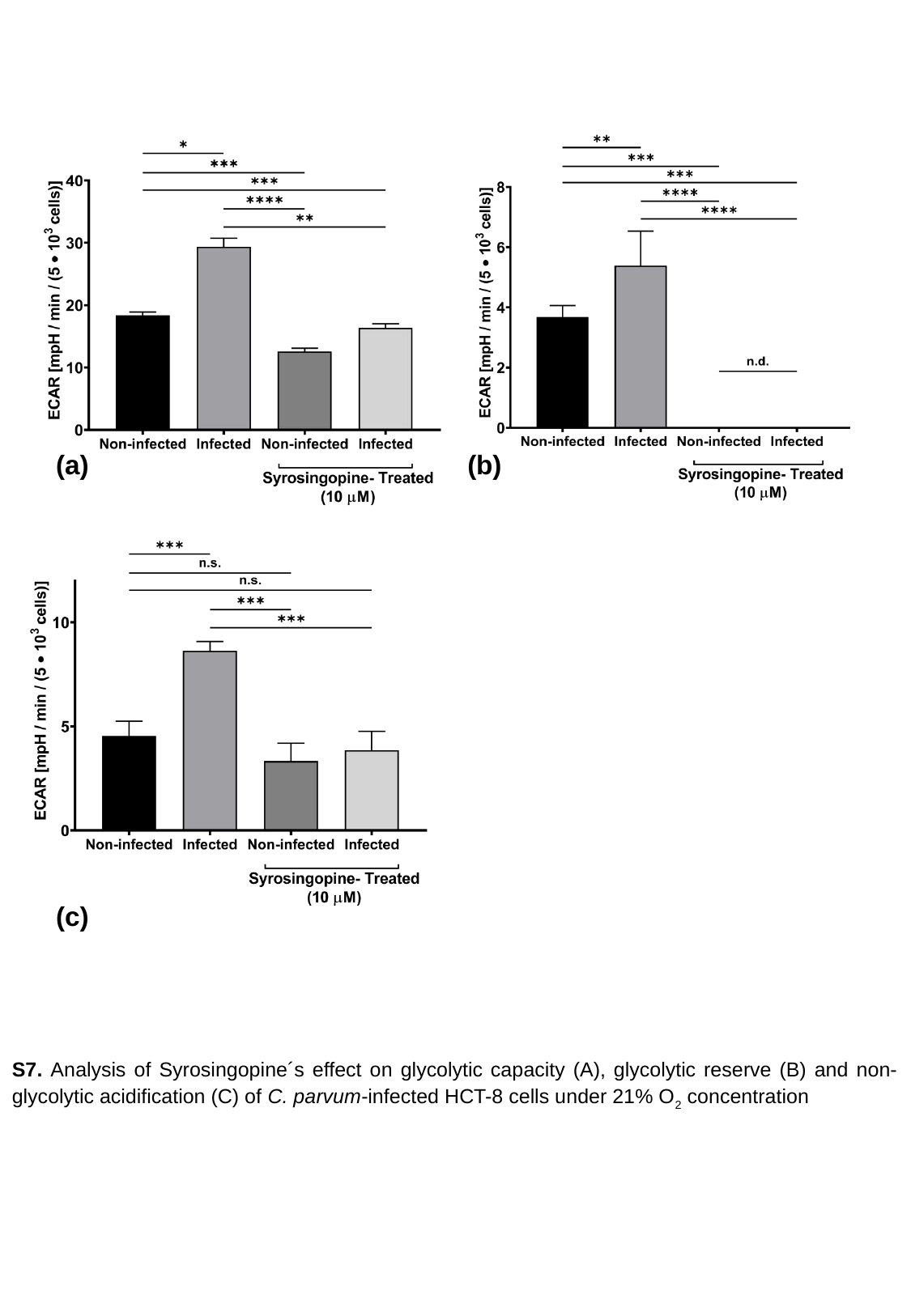

(a)
(b)
(c)
S7. Analysis of Syrosingopine´s effect on glycolytic capacity (A), glycolytic reserve (B) and non-glycolytic acidification (C) of C. parvum-infected HCT-8 cells under 21% O2 concentration

## Slide 8
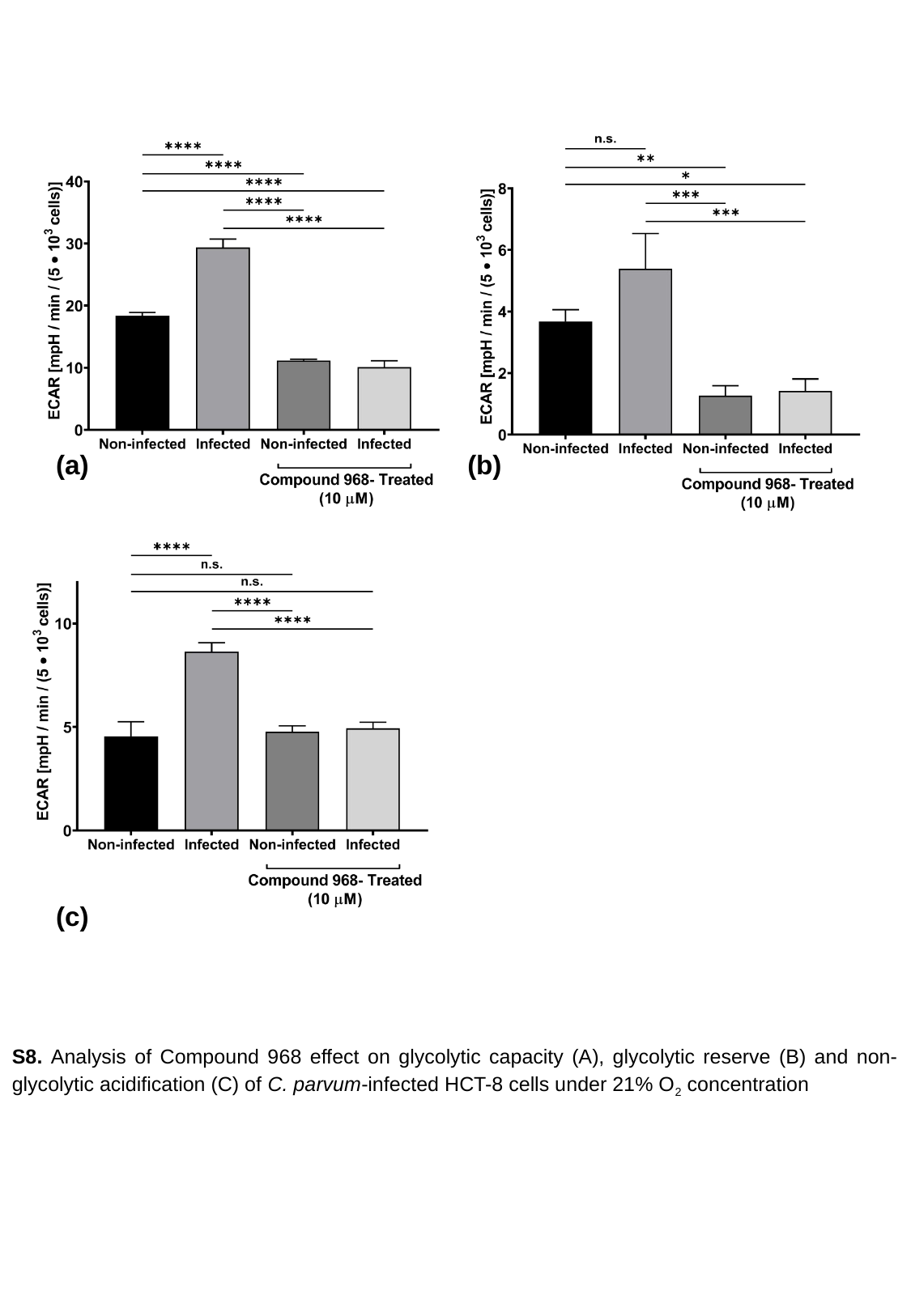

(a)
(b)
(c)
S8. Analysis of Compound 968 effect on glycolytic capacity (A), glycolytic reserve (B) and non-glycolytic acidification (C) of C. parvum-infected HCT-8 cells under 21% O2 concentration
